# Supplementary material for: Generating biomembrane-like local curvature in polymersomes via dynamic polymer insertion
Source: Nat Commun. 2021 Apr 14;12:2235. doi: 10.1038/s41467-021-22563-9 (PMC8046815; doi:10.1038/s41467-021-22563-9)
Supplement: Supplementary file 1 — Supplementary Information [file 41467_2021_22563_MOESM1_ESM.pdf]

Supplementary Information

Generating Biomembrane-like Local Curvature in Polymersomes via Dynamic Polymer Insertion

Jiawei Sun, Sjoerd J. Rijpkema, Jiabin Luan, Shaohua Zhang, Daniela A. Wilson*

Institute for Molecules and Materials Radboud University Heyendaalseweg 135, 6525 AJ, Nijmegen, the Netherlands

d.wilson@science.ru.nl

Contents

S1.Materials 4

S2. Experimental Procedures: 5

S2.1. Synthesis of poly(ethylene glycol)-polystyrene block copolymer: 5

S2.2 Temperature control experiment with the addition of PEG $_{2k}$ and PNIPAm$_{10k}$. 5

S2.3 NMR spectroscopy Analysis 6

S2.4 Synthesis of polystyrene 6

S2.5 PNIPAm dissociation determined by fluorescence spectroscopy. 6

S2.6 NMR Assignment 7

S3. Supplementary Figures and Tables 8

Supplementary Figure 1. Area difference of polymersome between two monolayers (ΔA) 8

Supplementary Figure 2. Cryo-TEM images of polymersome curvature formation 9

Supplementary Figure 3. Temperature control experiment of the insertion of PNIPAm 10

Supplementary Figure 4. Cryo-TEM images of nonaxisymmetric polymersomes 11

Supplementary Figure 5. Visualization of the PNIPAM distribution in the polymersome membrane by staining 12

Supplementary Figure 6 Staining oblates polymersomes with copper sulfate ($CuSO_4$) 13

Supplementary Figure 7 ^{1}H NMR spectra (400 MHz, 298 K) in D_2O of polymersomes. 14

Supplementary Figure 8 ^{1}H NMR spectra (400 MHz, 298 K) in D_2O of polymersomes. 15

Supplementary Figure 9 ^{1}H NMR spectra (400 MHz, 298 K) of PNIPAm$_{10k}$ 16

Supplementary Figure 10 ^{1}H NMR diffusion spectra (400 MHz, 298 K) in D_2O of PNIPAm$_{10k}$. 17

Supplementary Figure 11 ^{1}H NMR diffusion spectra (400 MHz, 298 K) in D_2O of polymersomes with free PNIPAm$_{10k}$ added. 18

Supplementary Figure 12 ^{1}H NMR diffusion spectra (400 MHz, 298 K) in D_2O of polymersomes with PNIPAm$_{10k}$ inserted. 19

Supplementary Figure 13. Staining of PNIPAm distribution in polymersome membrane 20

Supplementary Figure 14. Shape changes of polymersomes during slow addition of organic solvent. 21

Supplementary Figure 15. The shape transformation of polymersomes with the addition of PNIPAm 22

Supplementary Figure 16. Donor (polymersomes-Cy3) to acceptor (PNIPAm-Cy5) fluorescence energy transfer (FRET) demonstrating during the PNIPAm-membrane interaction. 23

Supplementary Figure 17. PNIPAm dissociation determined followed by Cy3-BHQ2 using fluorescence spectroscopy. 24

Supplementary Figure 18. Size distribution of PNIPAm particles. 25

Supplementary Table 1. The determined LCST of PNIPAm under different VFC. 26

S4. Supplementary References 26

S1.Materials

Unless and otherwise stated, all reagents and chemicals were purchased from commercial sources and used as received. Styrene (Sigma-Aldrich) was distilled to remove the inhibitor before polymerization. CuBr (Sigma-Aldrich) for ATRP was stirred with glacial acetic acid followed by washing with ethanol and diethyl ether and protected under Ar. Tetrahydrofuran (THF) for reaction was distilled under Argon from sodium/benzophenone. MilliQ water obtained from MilliQ QPOD purification system (18.2 MΩ) was used for self-assembly and quenching of samples withdrawn during the addition of solvent. α-methoxy-ω-hydroxypoly(ethylene glycol) (M_n 2000 g/mol), L (+) ascorbic acid, magnesium sulfate, sodium bicarbonate, sodium chloride, ethylenediaminetetraacetic acid (EDTA), 1-phenyl-1-trimethylsiloxyethene, α-bromoisobutyryl bromide, chloroform-d ($CDCl_3$), tert-butyl α-bromoisobutyrate and N,N,N',N'',N''-Pentamethyldiethylenetriamine (PMDETA) were purchased from SigmaAldrich. THF and anisole were obtained from Acros. MeOH, triethylamine and hydrogen peroxide (H_2O_2) was purchased from J.T. Baker. Diethyl ether (Carlo erba Reagents), 1,4- dioxane (Biosolve BV), dichloromethane (CH_2Cl_2) from Thermo Fisher Scientific were also used. THF and Dioxane used for DLS were inhibitor free for HPLC (≥99.9%) purchased from Sigma-Aldrich $CuSO_4{\cdot}5H_2O$ were also purchased from Sigma-Aldrich,Cyanine3/5 (cy3/5) amine was purchased from Lumiprobe GmbH, and Black Hole Quencher 2 (BHQ2) amine were purchased from Immunosource. Nuclear Magnetic resonance (NMR) characterization was carried out on a Bruker AVANCE HD nanobay console with a 9.4 T Ascend magnet (400 MHz) and a Bruker AVANCE III console with a 11.7 T UltraShield Plus magnet (500 MHz) equipped with a Bruker Prodigy cryoprobe, in $CDCl_3$ or D_2O. NMR spectra were recorded at 298 K unless otherwise specified. Chemical shifts are given in parts per million (ppm) with respect to tetramethylsilane (TMS, δ 0.00 ppm) in $CDCl_3$ or water (δ 4.79 ppm) in D_2O as internal standard for ^1H NMR. Coupling constants are reported as *J* values in Hz. Peak assignment where required is based on 2D $^1H-^{13}C$ gHSQCED spectra. Diffusion spectra were measured sequential. Fluorescence Spectra were recorded by JASCO

FP-8300ST Spectrofluorometer, using excitation at 512 nm and emission from 550-700 nm, with following settings have been used for all measurements: increment = 1 nm, integration time = 0.2 s, bandpass(emission) = bandpass(excitation) =2.5 nm.

S2. Experimental Procedures:

S2.1. Synthesis of poly(ethylene glycol)-polystyrene block copolymer: PEG-*b*-PS was synthesized according to our previous work[1]. Briefly, Poly(ethylene glycol) methyl ether (5.00 g, 2.50 mmol) was dried by co-evaporation with toluene, and then dissolved in freshly distilled THF in a flamed-dried Schlenk flask. After adding triethylamine (1.04 mL, 7.50 mmol), the mixture was cooled to 0 °C, followed by the addition of α-bromoisobutyryl bromide (616 mL, 5.00 mmol) dropwise. The solution was then stirred for 24 h while slowly warming to room temperature. The solution was concentrated after the white precipitate was filtered off. The polymer was then precipitated in ice cold diethyl ether (3x) and characterized by ^{1}H-NMR in $CDCl_3$. After synthesizing the initiator, CuBr (45 mg, 0.32 mmol) was added in the Schlenk tube and kept under vacuum for 15 min. After refilling with Ar, PMDETA (66 mL, 0.32 mmol) in anisole (0.5 mL) was added, followed by 15 min vigorously stirring. Styrene (5 mL, 43.6 mmol) in anisole (0.5 mL) was added via a syringe. The solution was cooled down to 0 °C and PEG-initiator (215 mg, 0.1 mmol) was added. The Schlenk tube was transferred into an oil bath at 90 °C. ^{1}H-NMR was used for monitoring the reaction process. Upon attainment of the required molecular weight, 1-phenyl-1- trimethylsiloxyethene (1.91 mL, 9.28 mmol) was added to quench the polymerization. The solution was diluted with CH_2Cl_2 and extracted with an aqueous EDTA solution (65 mM). The organic layer was collected and dried with $MgSO_4$ and concentrated. The polymer was obtained after precipitation in MeOH (3x), and dried under vacuum overnight and characterized by ^{1}H-NMR in $CDCl_3$ and GPC. The amphiphilic polymer obtained, PEG_{44}-b-PS_{172} had an number average molecular weight (M_w) of 19913.3 g/mol and a PDI of 1.04.

S2.2 Temperature control experiment with the addition of PEG_{2k} and $PNIPAm_{10k}$.

Firstly, 490 μL rigid polymersome solution was transferred into a 5 mL vial in an ice bath (0°C),

100 μg of PNIPAm or PEG_{2k} were then added into the polymersome solutions respectively. After ten minutes of mixing, THF : dioxane (4:1 v/v) mixture was added via syringe pump under a stirring speed of 900 rpm (Ika Stirrer, Germany) by using stirring plate with the rate of 300 μL h^{-1}. Samples were withdrawn and quenched while in the bath and after the temperature is back to 21 °C as scheduled and examined by TEM.

S2.3 NMR spectroscopy Analysis. Polymersomes with PNIPAm inserted sample was fabricated by addition of 200 μg of PNIPAm in the polymersome solution. After 23.08% of organic solvent was added to the system, withdrawn samples were quenched with MilliQ water, and washed through centrifugation (10621×g, 10 min) for 5 times to remove the free PNIPAm in the system. 5 batches of samples were concentrated and suspended in D_2O for the measurement.

S2.4 Synthesis of polystyrene-cy3 and PNIPAm-cy5. HOOC-PS 4 (61 mg, 3 μmol) / HOOC-PNIPAm (30 mg, 3 μmol) and cy3 NH2 (2 mg, 3 μmol) / cy5 NH_2 (2 mg, 3 μmol) / BHQ2 NH_2 (2 mg, 3 μmol) were dissolved in DMF (2 mL), followed by DiPEA (2 μL, 11.3 μmol) added to it. The solution was then stirred at 0 °C for 10 min and PyBOP (4.5 mg, 8.5 μmol) was added. The reaction was left to warm slowly to room temperature while stirring for two days, reaction was followed by TLC. Samples were dried under vacuum overnight to remove the DMF and then dissolved in Ethyl acetate, centrifuged to remove the undissolved salt. The product was purified on a silica column using Ethyl acetate as eluent. PNIPAm-cy5 was separated by CH_2Cl_2 with 5% methanol, PNIPAm-BHQ2 was separated by acetone.

S2.5 PNIPAm dissociation determined by fluorescence spectroscopy. Firstly, polymersomes with PS-cy3 embedded was formed by dissolving 9 mg PEG-PS and 1 mg PS-cy3 in THF : dioxane (4:1 v/v), and followed by water addition and dialysis as described above. After the polymersomes-cy3 was formed, 490 μL rigid polymersomes were transferred into a 5 mL vial, 500 μg (10 μL) of PNIPAm-cy5/ PNIPAm-BHQ2 was then added into the polymersome solutions. After ten minutes of mixing, THF : dioxane (4:1 v/v) mixture was added via a syringe pump with the rate of 300 μL h^{-1} under a stirring speed of 900 rpm using

stirring plate (Ika Stirrer, Germany). Samples were quenched at 30min/1 hour, and washed by centrifugation at 10621×g for 5 times. The polymersome pellet was then dissolved in water and the fluorescence was measured by Fluorescence Spectra.

S2.6 NMR Assignment

NMR Assignment PEG_{44}-*b*-PS_n:

^1H NMR (400 MHz, $CDCl_3$) δ 7.24 − 6.86 (m, 3nH, PS arom. ortho and para), 6.84 − 6.28 (m, 2nH, PS arom. meta), 3.78 (t, *J* = 4.9 Hz, 2H, $COOCH_2CH_2$), 3.64 (br s, 176H, PEG), 3.57 − 3.53 (m, 2H, CH_3OCH_2), 3.50 (t, *J* = 5.0 Hz, 2H, $CH_3OCH_2CH_2$), 3.38 (s, 3H, CH_3O), 2.30 − 1.70 (m, PS backbone C*H*), 1.70 − 1.17 (m, PS backbone CH_2), 0.90 (s, 6H, $C(CH_3)_2$). ^{13}C NMR (101 MHz, $CDCl_3$) δ 145.3 (PS quat. arom.), 127.8 (PS arom. ortho and meta), 125.7 (PS arom. para), 70.6 (PEG), 59.0 (CH_3O), 44.1 (PS backbone CH_2), 40.4 (PS backbone *C*H).

NMR Assignment $PNIPAm_n$:

^1H NMR (500 MHz, $CDCl_3$) δ 6.97 − 5.77 (bs, 1nH, N*H*), 4.01 (s, 1nH, $CH(CH_3)_2$), 2.55 − 1.42 (m, 3nH, backbone), 1.14 (s, 6nH, $CH(CH_3)_2$). ^{13}C NMR (125 MHz, $CDCl_3$) δ 42.3 (backbone *C*H), 41.4 ($CH(CH_3)_2$), 34.8 (backbone CH_2), 22.6 $CH(CH_3)_2$.

^1H NMR (500 MHz, D_2O) δ 3.83 (s, 1nH, $CH(CH_3)_2$), 2.20 − 1.79 (m, 1nH, backbone C*H*), 1.79 − 1.27 (m, 2nH, backbone CH_2), 1.08 (s, 6nH, $CH(CH_3)_2$). ^{13}C NMR (125 MHz, D_2O) δ 41.6 ($CH(CH_3)_2$), 42.6 (backbone *C*H), 34.7 (backbone CH_2), 21.5 $CH(CH_3)_2$.

S3. Supplementary Figures and Tables

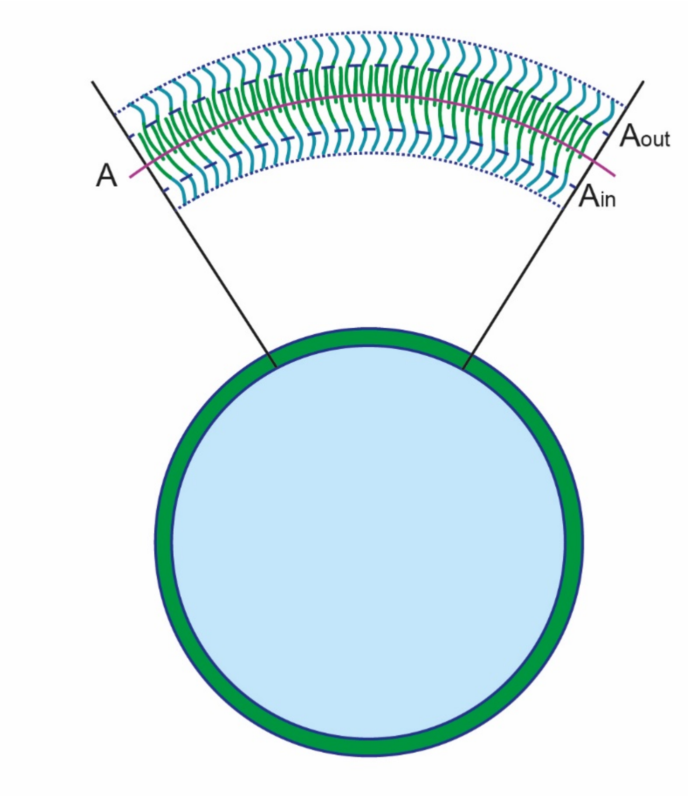

Supplementary Figure 1. Area difference of polymersome between two monolayers (ΔA). Three surface areas are defined in bilayer particles, the actual surface area A is determined by the neutral plane of the bilayer (red line). A_{out} means the outer surface area determined by the neutral planes of the outer monolayer, and A_{in} means the inner surface determined by the inner monolayer (blue dashed lines). The reduced monolayer area difference (Δa) is calculated by dividing the area difference ΔA of a vesicle by that of a sphere with the same surface area A[2, 3].

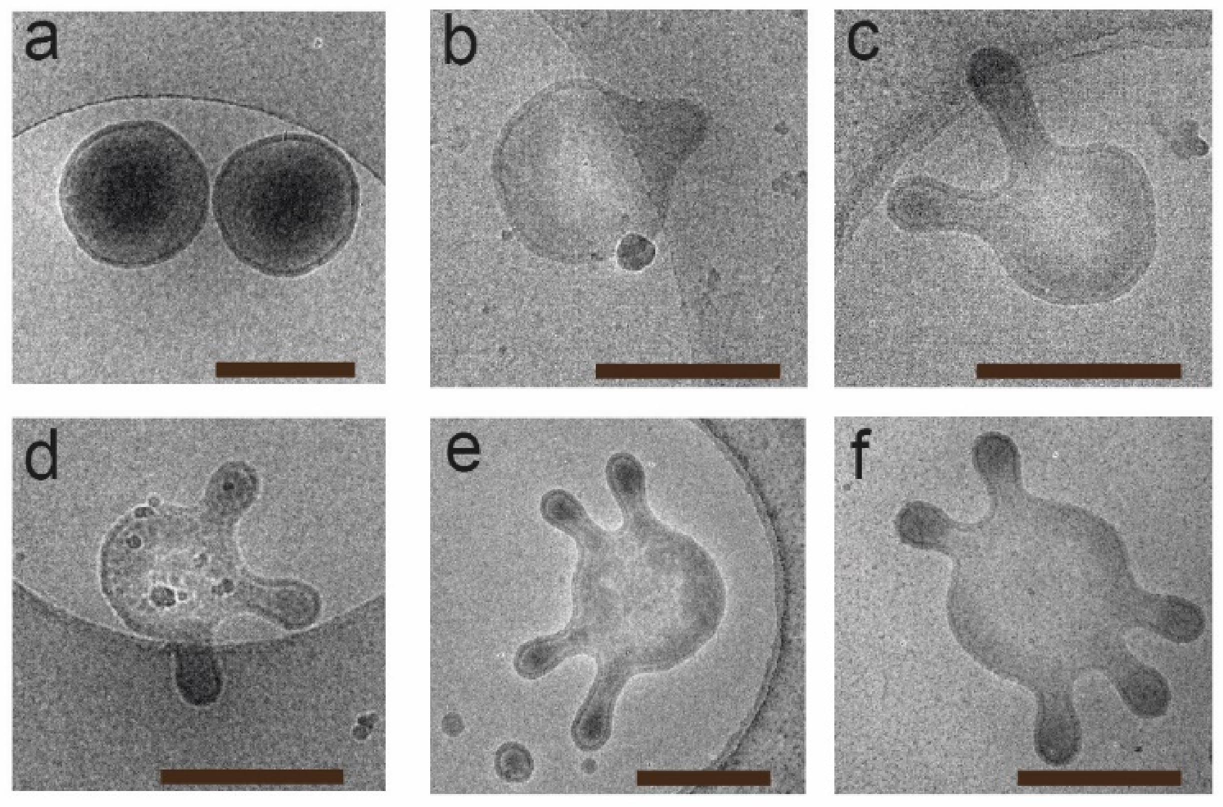

Supplementary Figure 2. Cryo-TEM images of polymersome curvature formation, after 5 µg (a), 10 µg (b), 25 µg (c), 50 µg (d), 100 µg (e), 200 µg (f) of PNIPAm was added. Scale bar 200 nm.

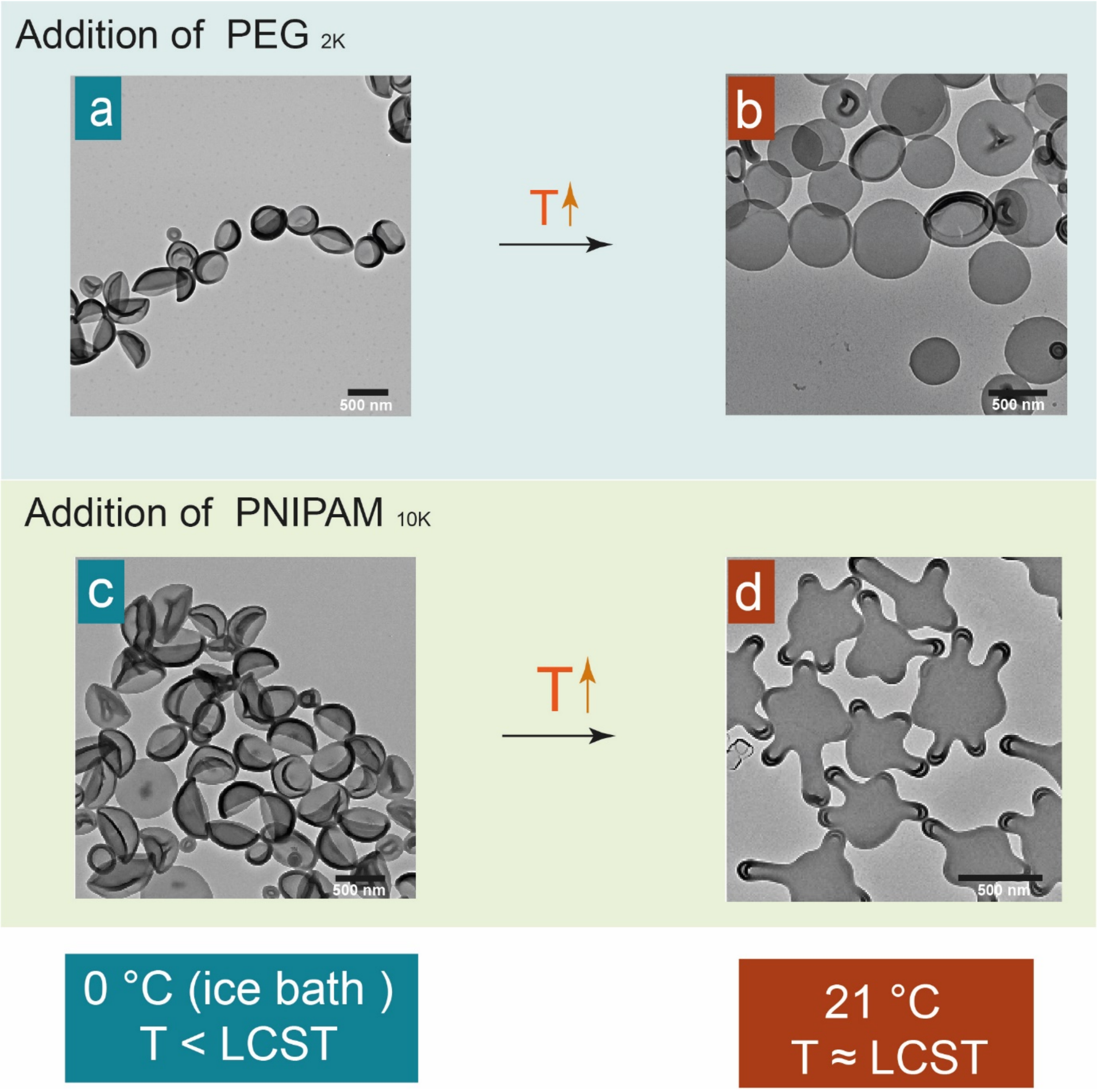

Supplementary Figure 3. Temperature control experiment of the insertion of PNIPAm. PEG 2k with no LCST transition was used for comparison. The transition of PNIPAm happened when the temperature was increased from 0 °C to 21°C near LCST of PNIPAm. Sample with the addition of 100 μg PEG_{2k} at 0 °C with 23.07% organic solvent (a), and increase the temperature to 21 °C (b). With the addition of 100 μg $PNIPAm_{10k}$ at 0 °C with 23.07% organic solvent (c), and increase the temperature to 21 °C (d).

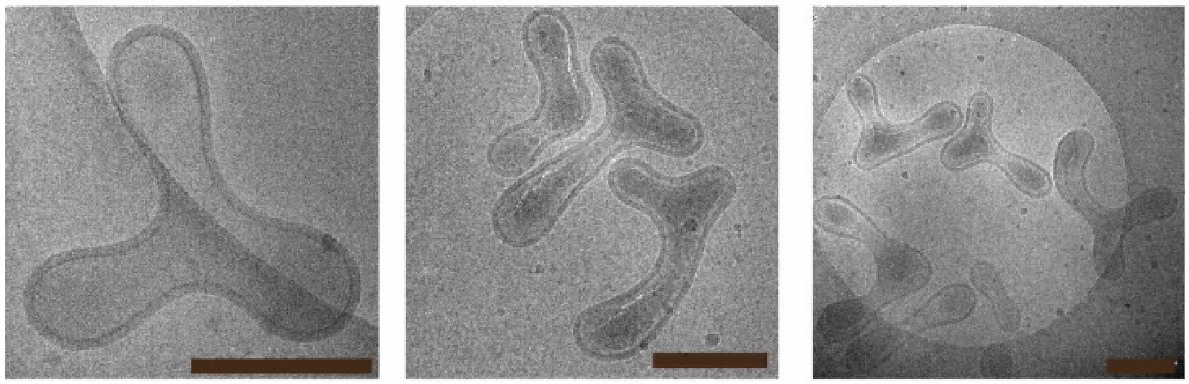

Supplementary Figure 4. Cryo-TEM images of nonaxisymmetric polymersomes, after more than 250 µg of PNIPAm was added, including cigar-like shape, rackets-like shape, boomerang-like shape, and 3-armed starfish-like shape. Scale bar 500 nm.

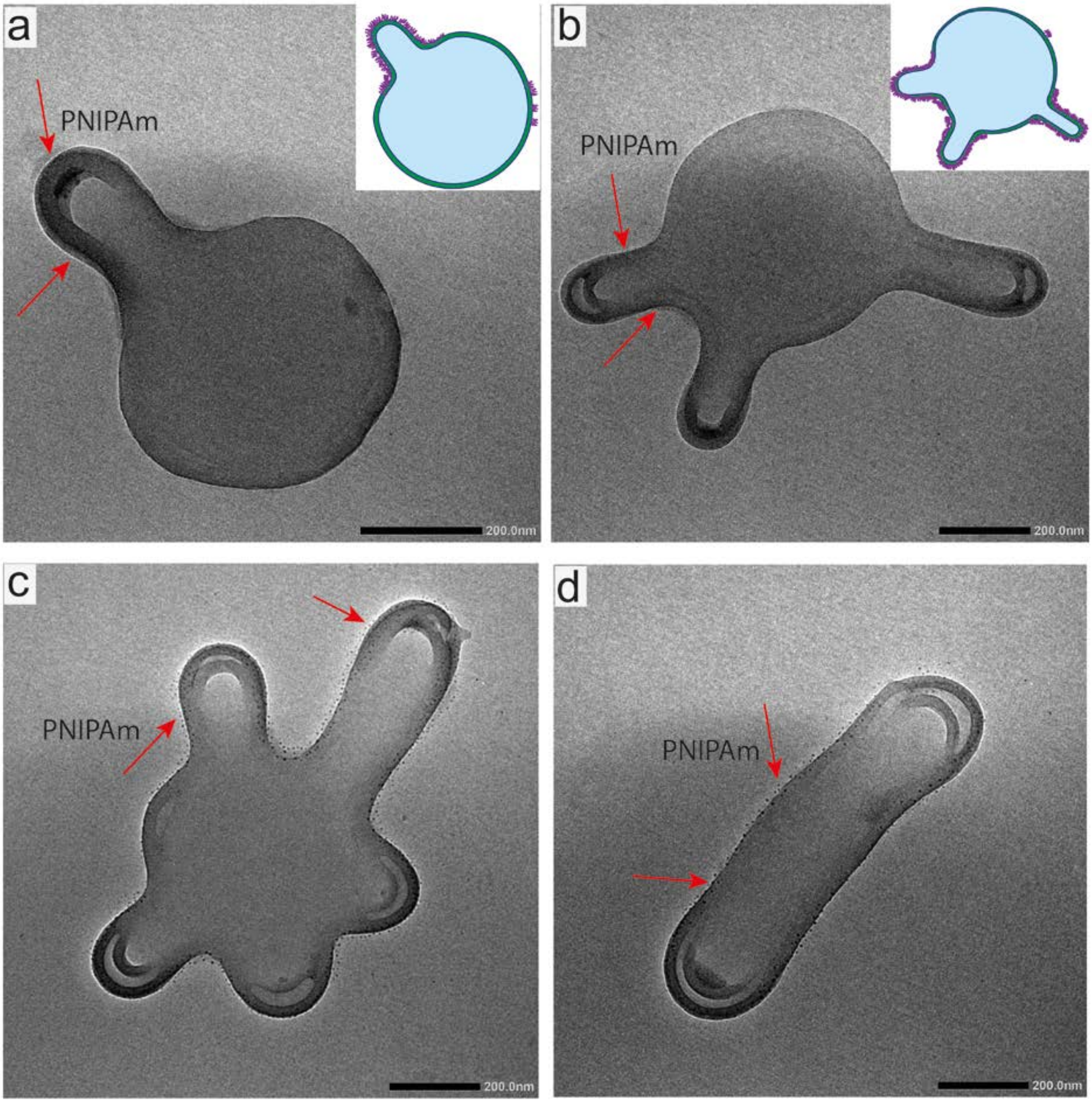

Supplementary Figure 5. Visualization of the PNIPAM distribution in the polymersome membrane by staining. TEM image of polymersome membrane dyed with copper sulfate ($CuSO_4$), coordination of Cu^{2+} with PNIPAm makes this polymer visible, red arrows pointed towards the PNIPAm presented as black dots, one tentacle polymersome(a), three tentacles polymersome (b), five tentacles polymersome (c), cigar-like polymersomes (d). Scale bar 200 nm.

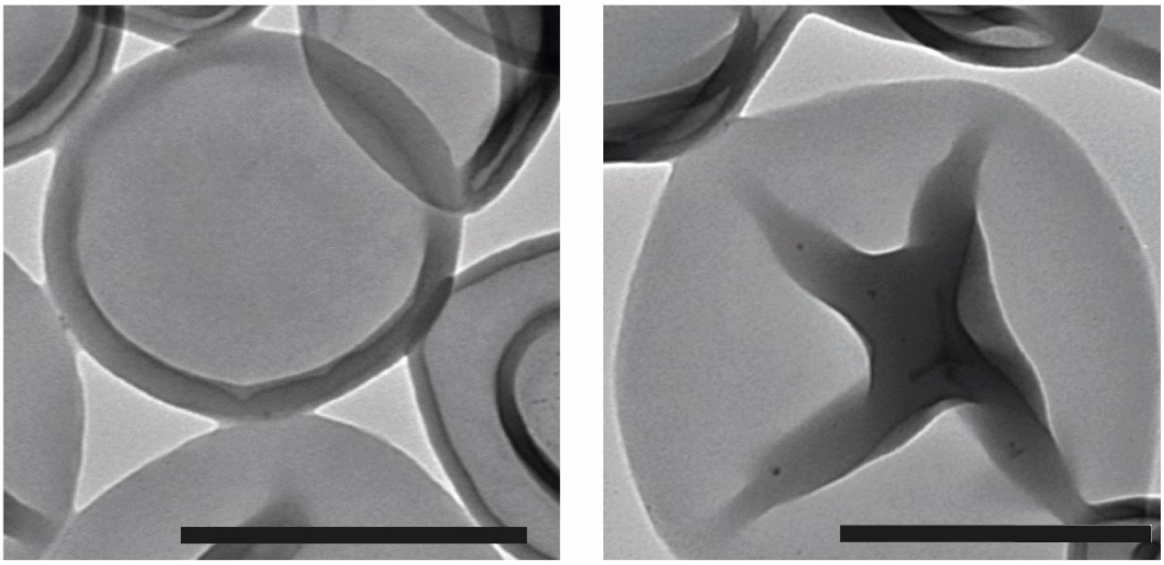

Supplementary Figure 6 Staining oblates polymersomes with copper sulfate ($CuSO_4$). No black dot was observed in or near the membrane. Scale bar 500 nm

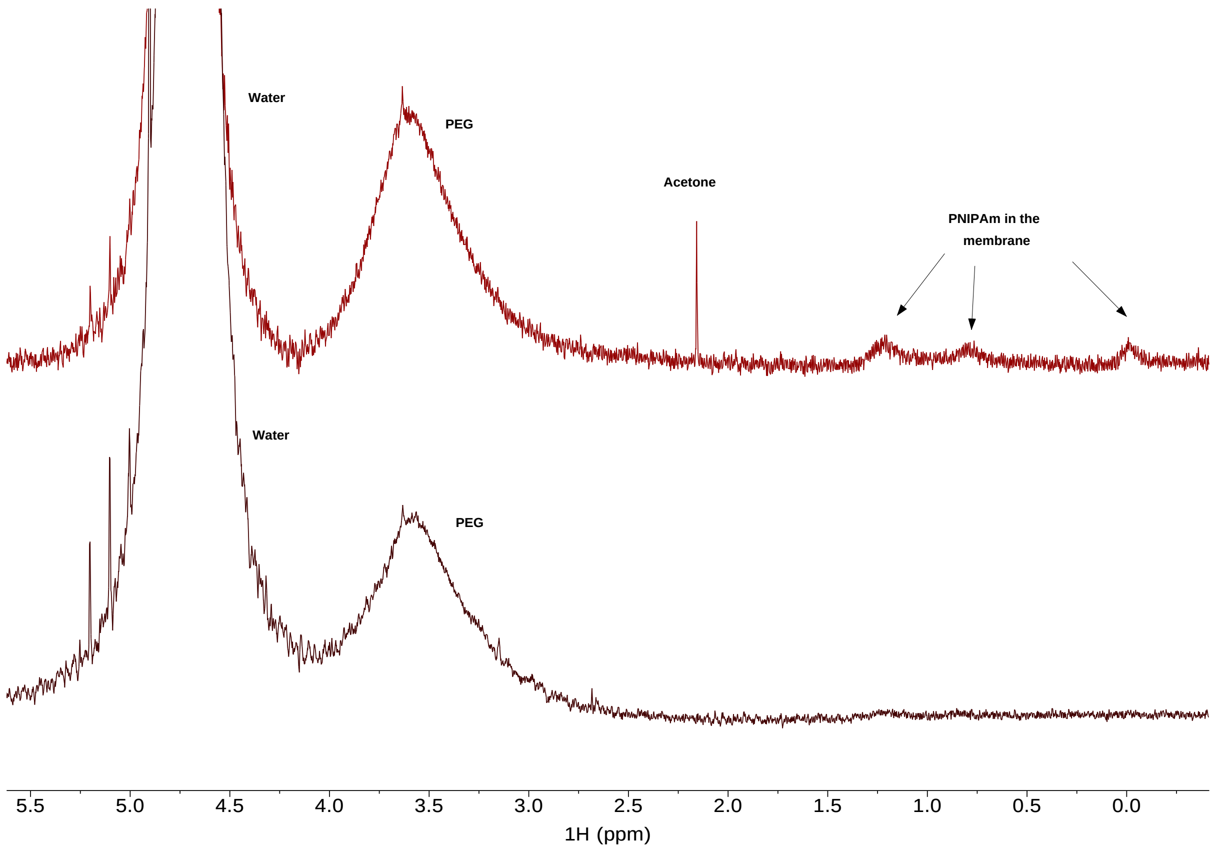

Supplementary Figure 7 ^{1}H NMR spectra (400 MHz, 298 K) in D_2O of polymersomes, with (top) and without (bottom) PNIPAm inserted. A broad signal from the PEG at the surface of the polymersomes is observed between 4.0 and 3.0 ppm. Similar broad signals can be observed at 1.2, 0.8 and 0.0 ppm in the top spectrum, which might originate from the PNIPAm. The broadness of the peaks indicate insertion in the membrane. These signals are missing when the polymersomes are prepared without PNIPAm.

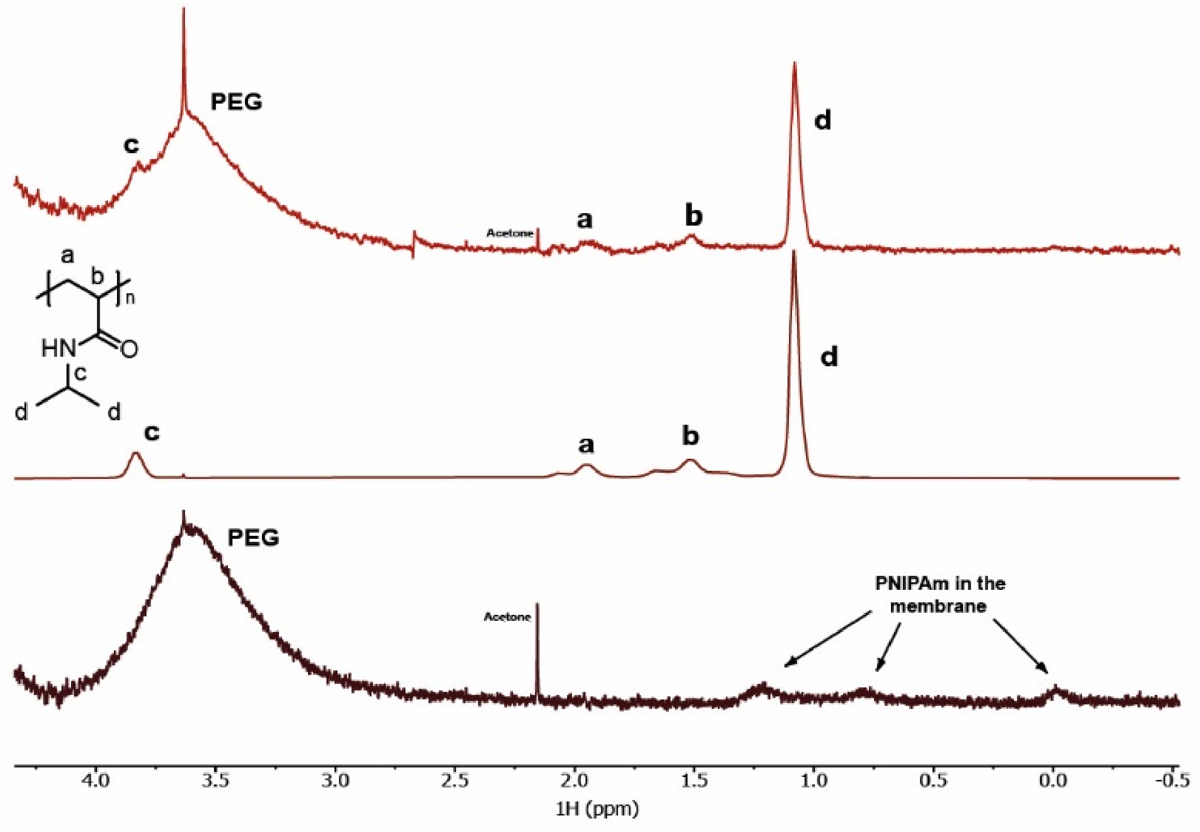

Supplementary Figure 8 ^{1}H NMR spectra (400 MHz, 298 K) in D_2O of polymersomes. With free PNIPAm added (top), free PNIPAm (middle) and polymersomes with inserted PNIPAm (bottom). A broad signal from the PEG is observed between 4.0 and 3.0 ppm, together with a sharp peak at 3.63, representing PEG at and sticking out of the surface of the polymersomes respectively. When PNIPAm is added in polymersomes without solvent addition, it cannot enter the membrane and the signal of free PNIPAm can be observed (top). When PNIPAm is added to polymersomes followed with solvent addition, it can enter the membrane, and presented as solid state, since broad signals can be observed at 1.2, 0.8 and 0.0 ppm. The change of microenvironment might cause the peak shift. Meanwhile, the signal of free PNIPAm is not observed since its washed over (bottom). The broadness of the peaks indicate insertion into the membrane.

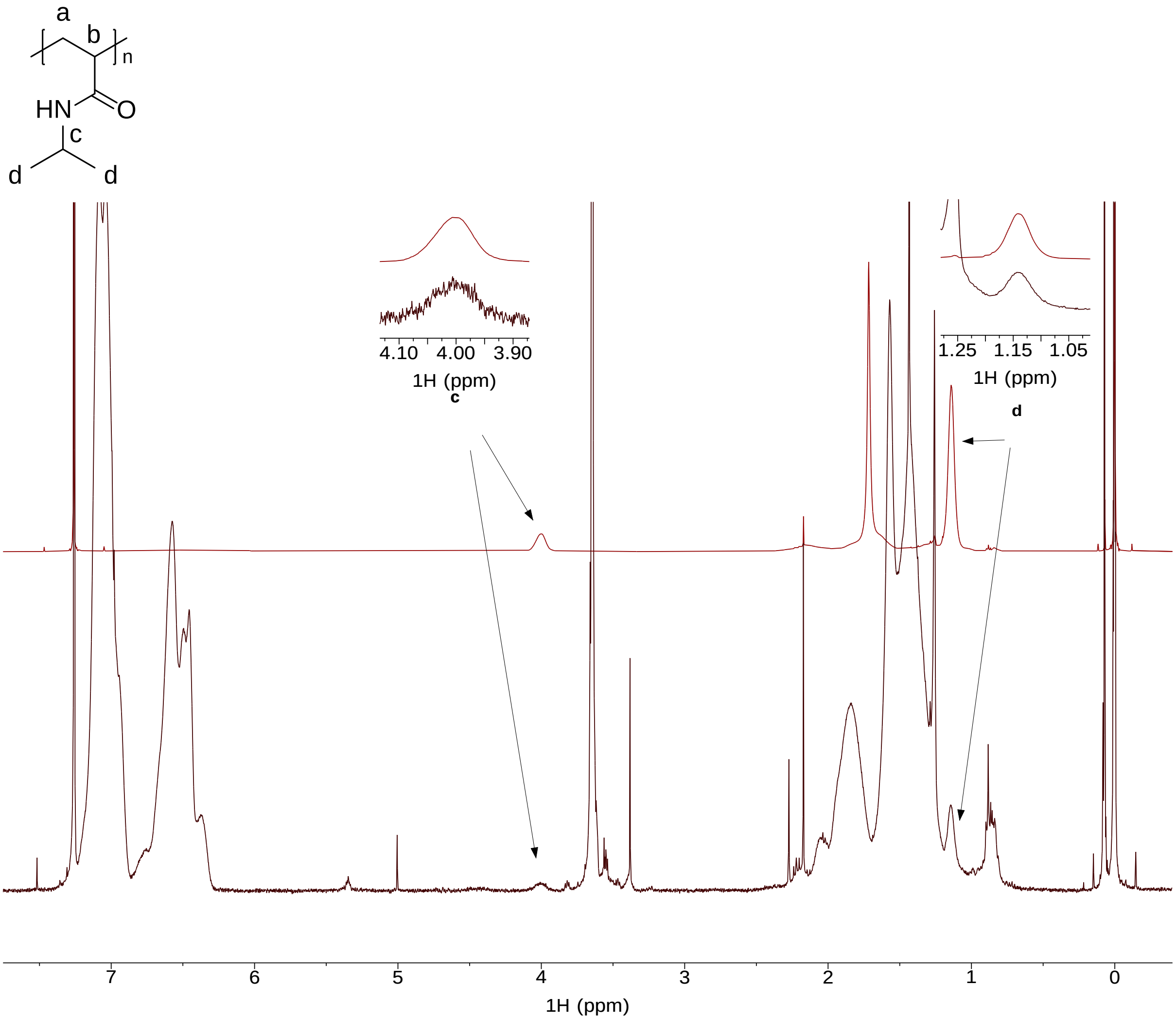

Supplementary Figure 9 ^{1}H NMR spectra (400 MHz, 298 K) of PNIPAm$_{10k}$. After freeze-drying the polymersomes with PNIPAm 10k inserted and re-dissolving in $CDCl_3$ (bottom), and free PNIPAm 10k in $CDCl_3$ (top) for comparison. PEG-*b*-PS is predominantly observed, as it made up the membrane of the polymersome. A small amount of PNIPAm can also be observed by the signals from the branches (c and d). The backbone (a and b) is not clearly visible, as it overlaps with the backbone of the PS. The PNIPAm (10k Da, n=89) peak at 4.0 ppm integrates to 3.18 compared to the PEG-*b*-PS, indicating around 3.6% PNIPAm compared to the molar amount of PEG-*b*-PS (10 mg, 0.45 µmol) in the sample. The total amount of PNIPAm inserted is 0.0162 µmol, 0.16 mg.

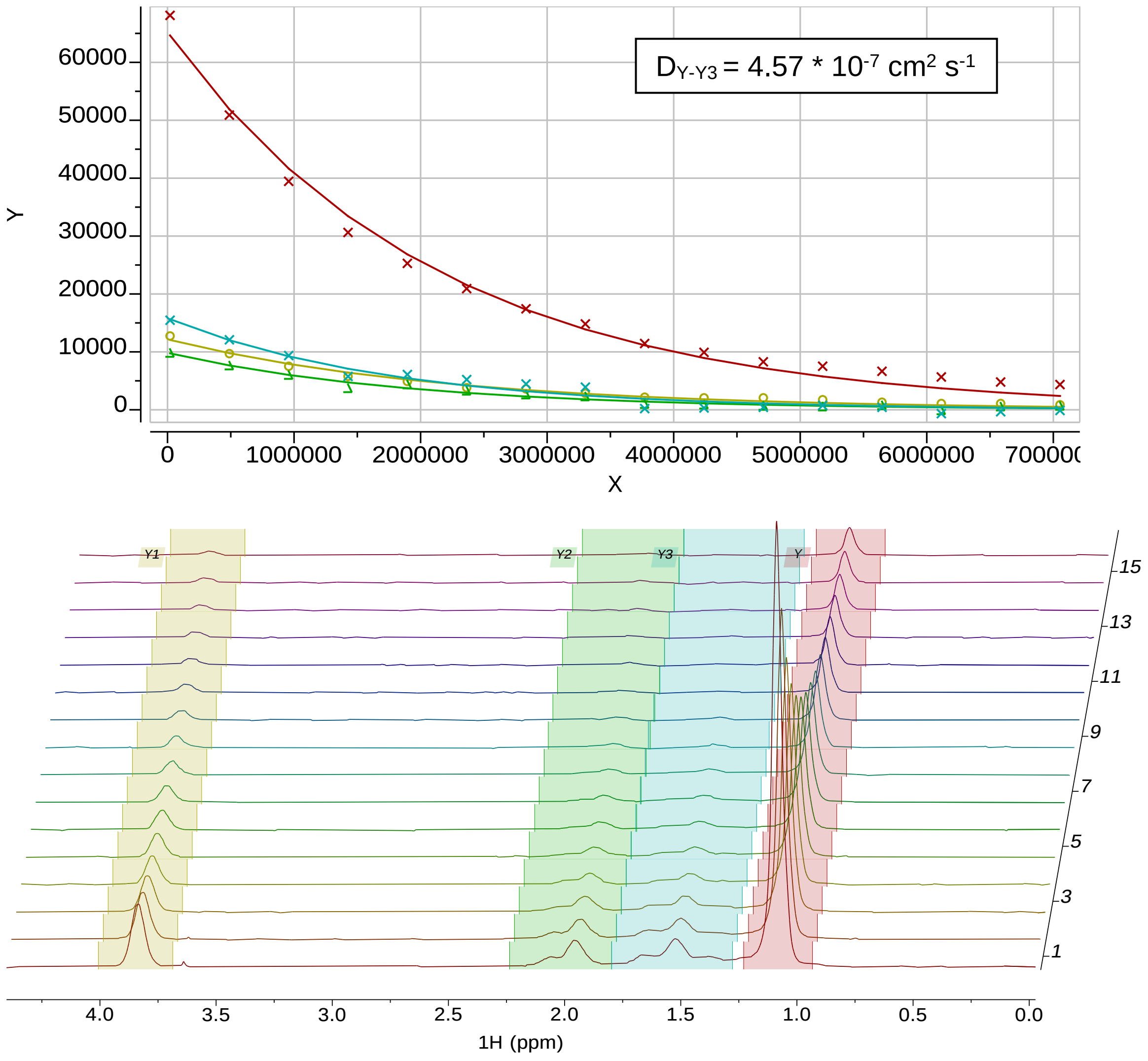

Supplementary Figure 10 ^{1}H NMR diffusion spectra (400 MHz, 298 K) in D_2O of PNIPAm$_{10k}$. Gradient strengths ranged from 5% to 95%. The absolute gradient strength was not calibrated prior to use as a relative comparison was all that was desired. DY-Y3 is the average diffusion coefficient of the PNIPAm signals.

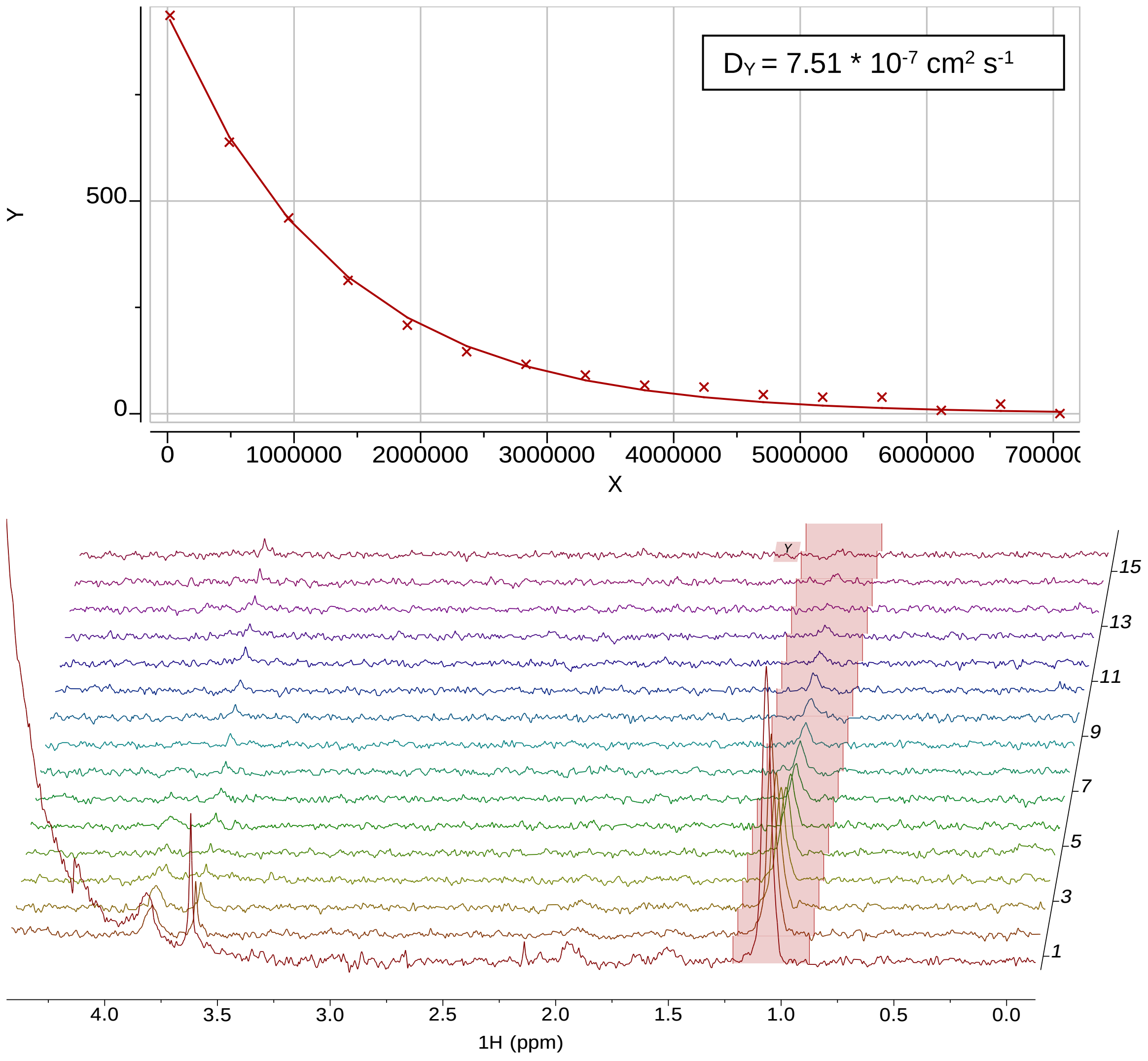

Supplementary Figure 11 ^{1}H NMR diffusion spectra (400 MHz, 298 K) in D_2O of polymersomes with free $PNIPAm_{10k}$ added. Gradient strengths ranged from 5% to 95%. The absolute gradient strength was not calibrated prior to use as a relative comparison was all that was desired. DY is the diffusion coefficient of the two methyl groups in PNIPAm. The other signals were not used due to their low signal to noise or overlap with the water peak. The diffusion coefficient is in the same order of magnitude compared to free PNIPAm 10k, indicating it is still free in solution. The slight variation in diffusion speed can be explained by a difference in viscosity between pure D_2O and a polymersome suspension.

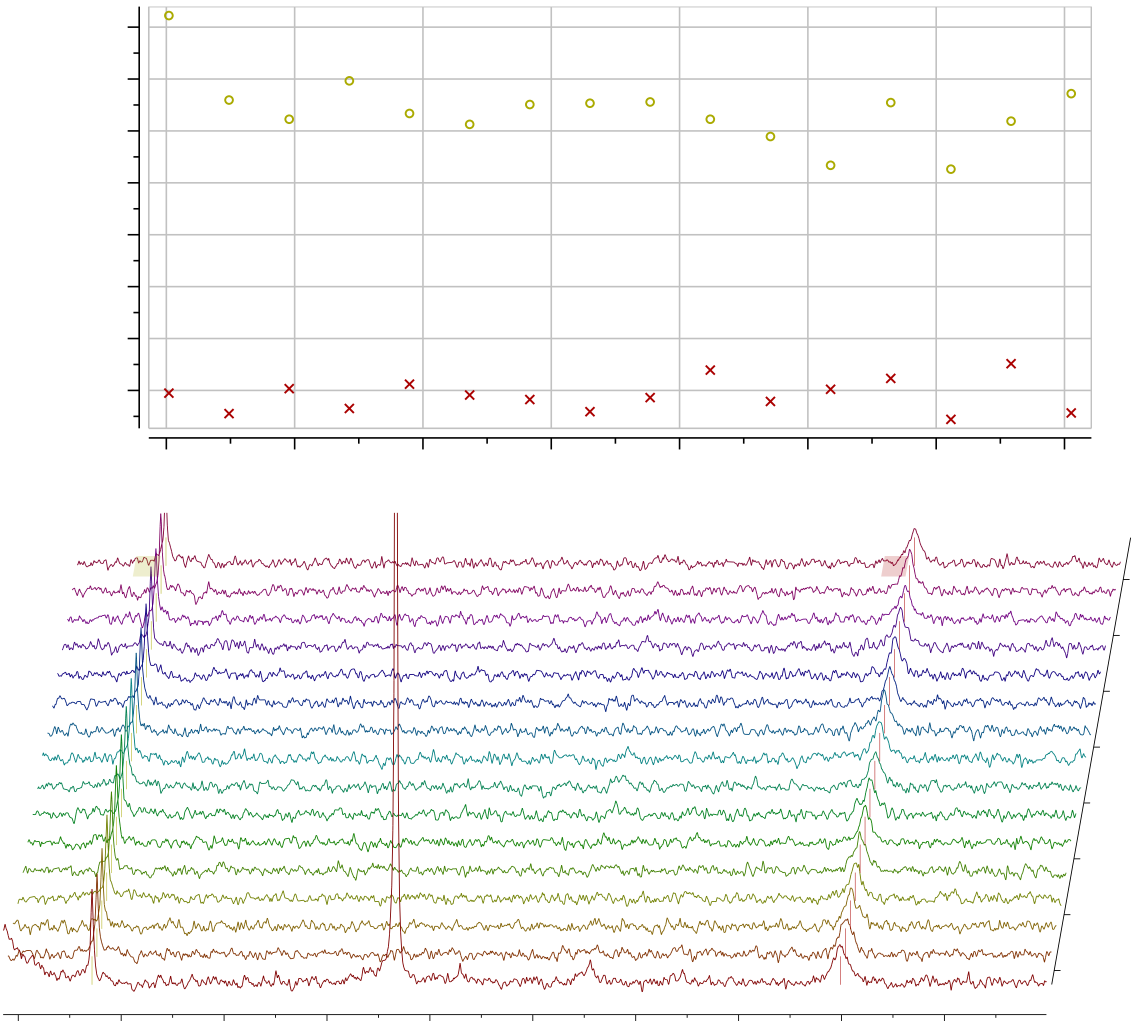

Supplementary Figure 12 ^{1}H NMR diffusion spectra (400 MHz, 298 K) in D_2O of polymersomes with PNIPAm$_{10k}$ inserted. Gradient strengths ranged from 5% to 95%. The same gradient strength was used as with free PNIPAm. The absolute gradient strength was not calibrated prior to use as a relative comparison was all that was desired. Similar to PEG, no visible diffusion of PNIPAm was observed, despite having the same weight, indicating the PNIPAm is trapped in the membrane of the polymersomes, slowing it down extremely.

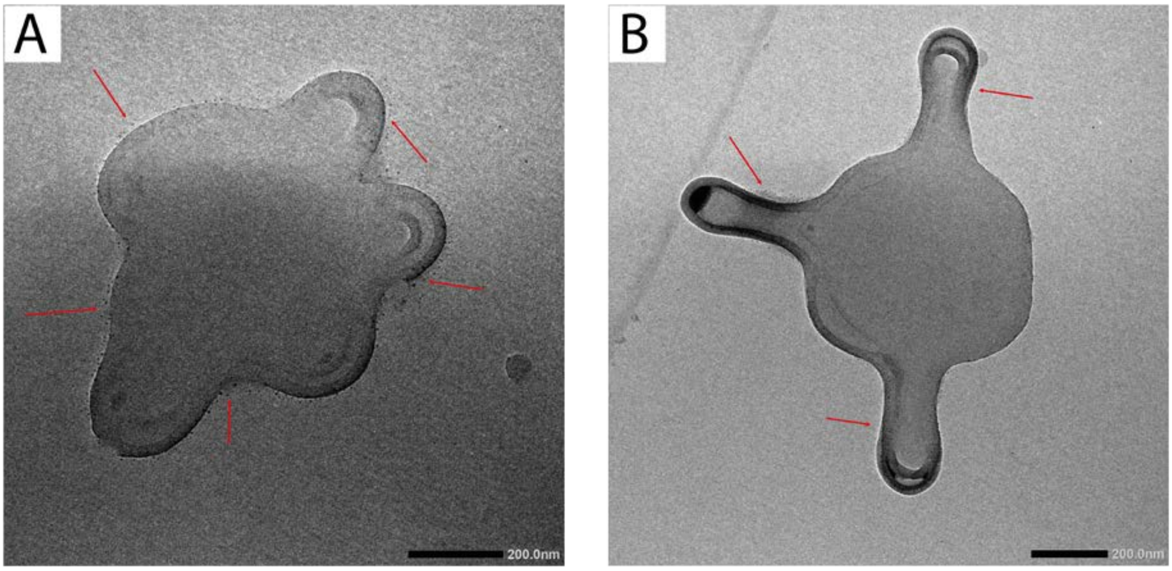

Supplementary Figure 13. Staining of PNIPAm distribution in polymersome membrane. TEM image of polymersome with the addition of 100 μg PNIPAm at 23.08% VFC, and membrane dyed with copper sulfate ($CuSO_4$) at different time point (a) 0 min and (b) 20 min. Scale bar 200 nm. PNIPAm was first everywhere on the membrane, and then moved to the protrusions on the membrane.

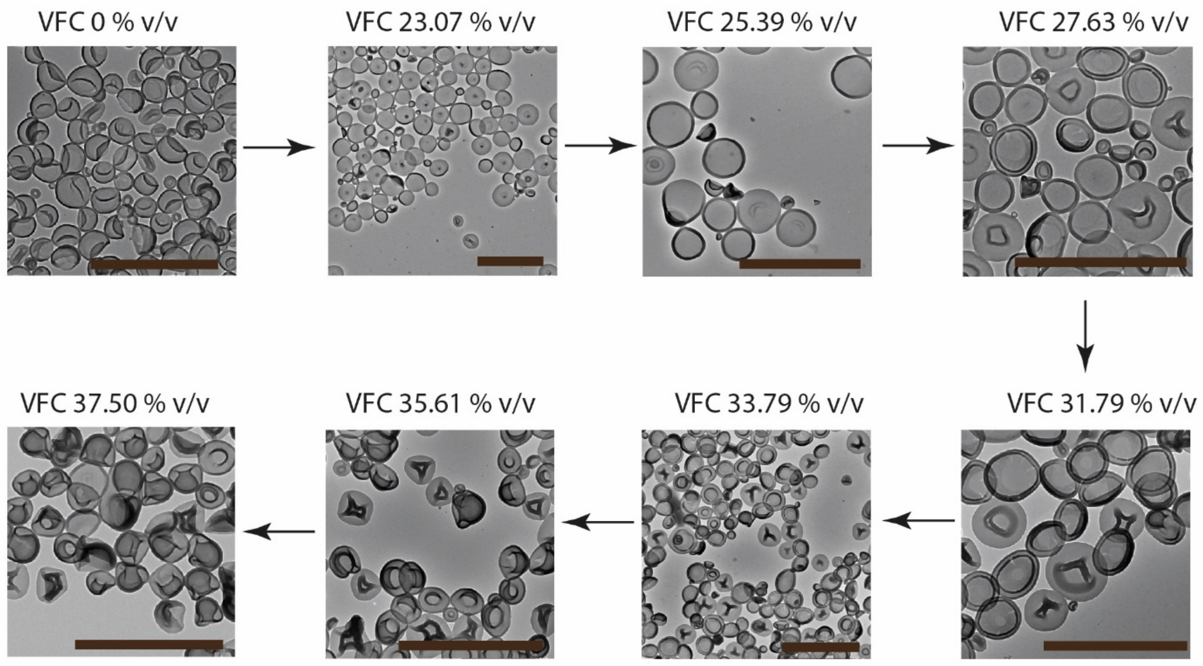

Supplementary Figure 14. Shape changes of polymersomes during slow addition of organic solvent. Samples were taken at different ratios of organic solvent. Scale bar 2 µm.

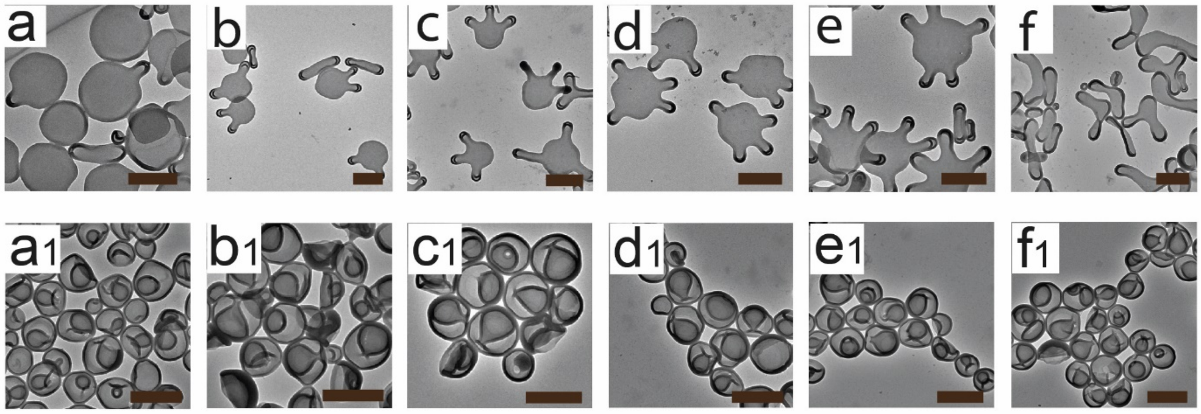

Supplementary Figure 15. The shape transformation of polymersomes with the addition of PNIPAm, from 10 µg (a), 25 µg (b), 50 µg (c), 100 µg (d), 200 µg (e) to 250 µg (f). At 23.07% organic solvent (a-f), polymersomes changed to different shapes, with 37.5% solvent added into to the samples, all of them changed to stomatocyte polymersomes (a1-f1). Scale bar 500 nm.

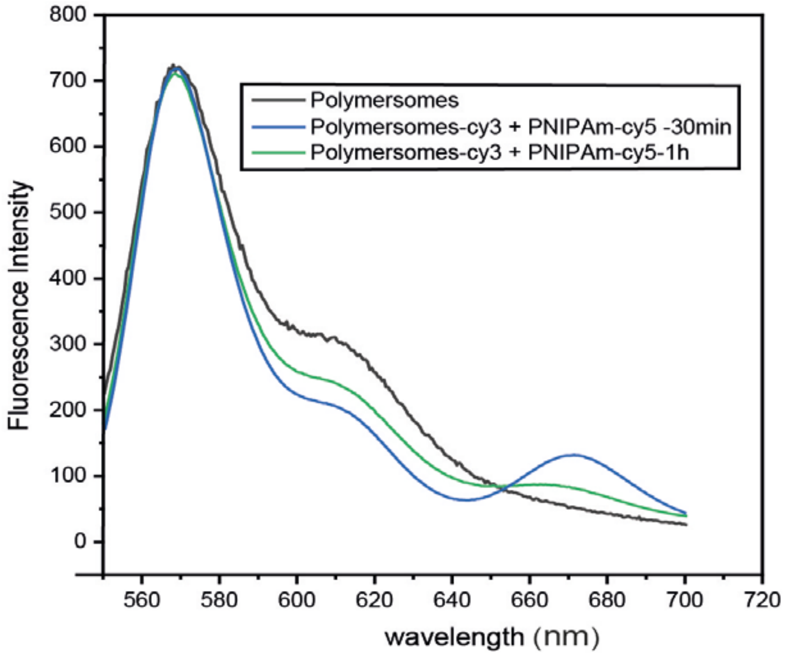

Supplementary Figure 16. Donor (polymersomes-Cy3) to acceptor (PNIPAm-Cy5) fluorescence energy transfer (FRET) demonstrating during the PNIPAm-membrane interaction. Cy5 fluorescence emission spectra (640-700 nm) obtained by excitation of Cy3 at 512 nm. After addition of 150 µl organic solvent to the system, the PNIPAm-Cy5 inserted into polymersome membrane. When Cy3 (embedded in the polymersome membrane during self-assembly) was excited at 512nm, the emission of Cy5 was also excited by the emission from Cy3 (blue line). After addition of 300 µl organic solvent to the system, the PNIPAm-Cy5 was slowly dissociated from polymersome membrane. When Cy3 was excited at 512nm, the emission of Cy5 was slightly excited by the emission from Cy3, but much lesser than 150 µl organic solvent addition (green line).

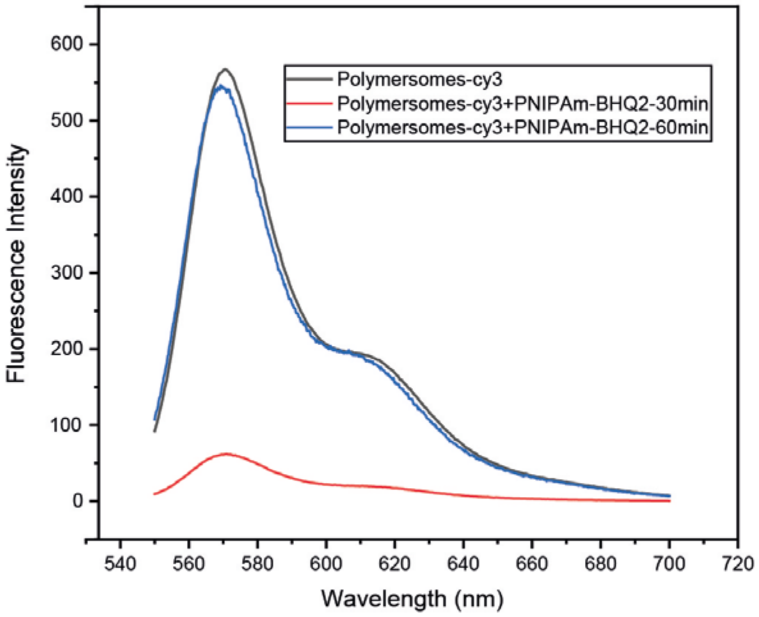

Supplementary Figure 17. PNIPAm dissociation determined followed by Cy3-BHQ2 using fluorescence spectroscopy. Cy3 fluorescence emission spectra obtained by excitation of Cy3 at 512 nm. After addition of 150 µl organic solvent to the system, the PNIPAm-BHQ2 inserted into polymersome membrane, the excitation of Cy3 was quenched by BHQ2 (red line). After addition of 300 µl organic solvent to the system, the PNIPAm-BHQ2 was slowly dissociated from polymersome membrane, the excitation of Cy3 regained (blue line).

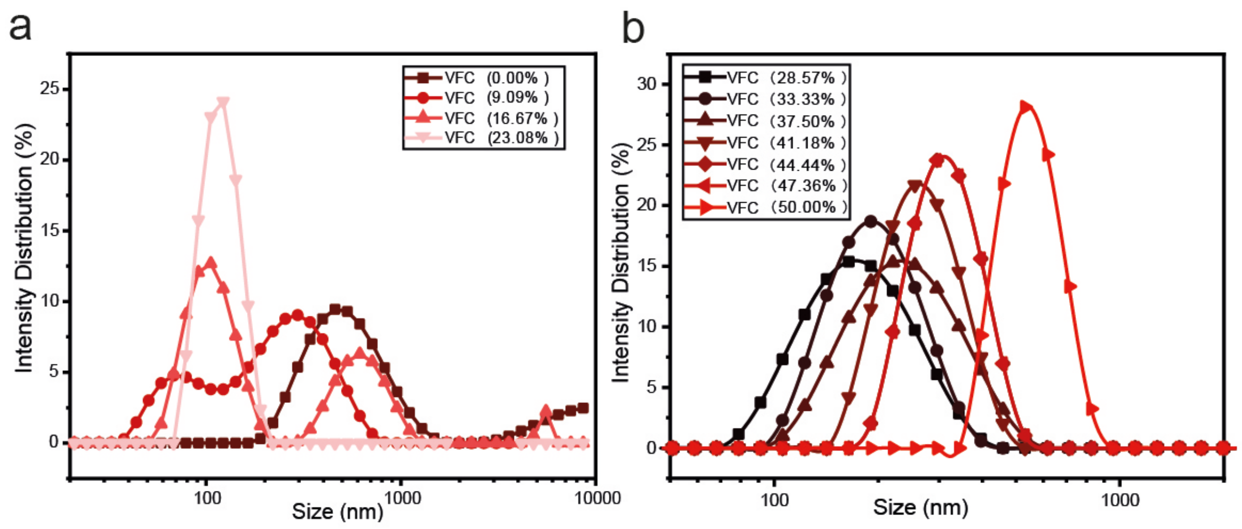

Supplementary Figure 18. Size distribution of PNIPAm particles. Organic solvent was slowly added into the PNIPAm water solution, from 0.00% to 23.07% (a), and 28.57% to 50.0% (b).

VFC %（v/v）	PNIPAm LCST
0	32.9174
9.09091	28.47873
16.66667	24.39262
23.07692	19.93986
28.57143	17.56691
33.33333	11.23474
37.5	9.17146
41.17647	7.58093

Supplementary Table 1. The determined LCST of PNIPAm under different VFC.

S4. Supplementary References

1. Wilson, D. A., Nolte, R. J. M.&van Hest, J. C. M. Autonomous movement of platinum-loaded stomatocytes. *Nat Chem* **4**(4), 268-274 (2012).

2. van Rhee, P. G. et al. Polymersome magneto-valves for reversible capture and release of nanoparticles. *Nat Commun* **5**(1), 5010 (2014).

3. Ziherl, P.&Svetina, S. Nonaxisymmetric phospholipid vesicles: Rackets, boomerangs, and starfish. *EPL* **70**(5), 690-696 (2005).
